# Role of AI in empowering and redefining the oncology care landscape: perspective from a developing nation

**DOI:** 10.3389/fdgth.2025.1550407

**Published:** 2025-03-04

**Authors:** Isha Goel, Yogendra Bhaskar, Nand Kumar, Sunil Singh, Mohammed Amanullah, Ruby Dhar, Subhradip Karmakar

**Affiliations:** ^1^ Department of Biochemistry, All India Institute of Medical Sciences (AIIMS), New Delhi, India; ^2^ Department of Psychiatry, All India Institute of Medical Sciences (AIIMS), New Delhi, India; ^3^ ICMR Computational Genomics Centre, Indian Council of Medical Research (ICMR), New Delhi, India; ^4^ Department of Clinical Biochemistry, College of Medicine, King Khalid University, Abha, Saudi Arabia

**Keywords:** artificial intelligence, digital health, cancer management, personalized medicine, developing nation

## Abstract

Early diagnosis and accurate prognosis play a pivotal role in the clinical management of cancer and in preventing cancer-related mortalities. The burgeoning population of Asia in general and South Asian countries like India in particular pose significant challenges to the healthcare system. Regrettably, the demand for healthcare services in India far exceeds the available resources, resulting in overcrowded hospitals, prolonged wait times, and inadequate facilities. The scarcity of trained manpower in rural settings, lack of awareness and low penetrance of screening programs further compounded the problem. Artificial Intelligence (AI), driven by advancements in machine learning, deep learning, and natural language processing, can profoundly transform the underlying shortcomings in the healthcare industry, more for populous nations like India. With about 1.4 million cancer cases reported annually and 0.9 million deaths, India has a significant cancer burden that surpassed several nations. Further, India's diverse and large ethnic population is a data goldmine for healthcare research. Under these circumstances, AI-assisted technology, coupled with digital health solutions, could support effective oncology care and reduce the economic burden of GDP loss in terms of years of potential productive life lost (YPPLL) due to India's stupendous cancer burden. This review explores different aspects of cancer management, such as prevention, diagnosis, precision treatment, prognosis, and drug discovery, where AI has demonstrated promising clinical results. By harnessing the capabilities of AI in oncology research, healthcare professionals can enhance their ability to diagnose cancers at earlier stages, leading to more effective treatments and improved patient outcomes. With continued research and development, AI and digital health can play a transformative role in mitigating the challenges posed by the growing population and advancing the fight against cancer in India. Moreover, AI-driven technologies can assist in tailoring personalized treatment plans, optimizing therapeutic strategies, and supporting oncologists in making well-informed decisions. However, it is essential to ensure responsible implementation and address potential ethical and privacy concerns associated with using AI in healthcare.

## 1 Introduction

### 1.1 Cancer care in India: challenges and solutions

According to The World Bank report, 2024, India has become the most populous country surpassing China in 2022, with over 1.417 billion inhabitants, the majority of whom were under 25 (1). The size and growing pace of the Indian population present various concerns. It puts an enormous strain on healthcare, education, infrastructure, environment, and employment opportunities. Providing proper healthcare services to such a large population is a significant challenge.

**Figure 1:**
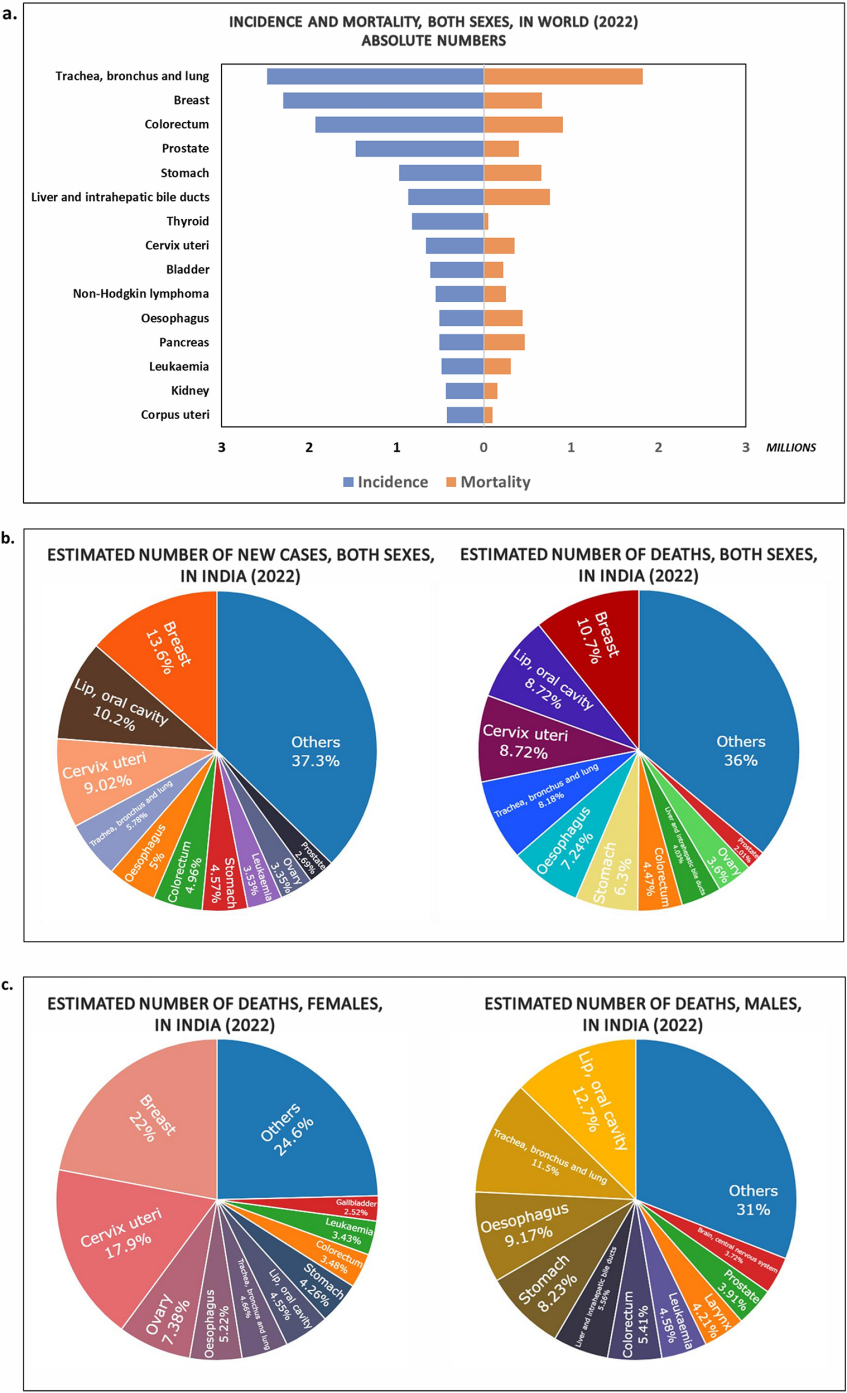
Charts showing the estimates of new cases and deaths due to different types of cancer during the year 2022 in **(a)** Worldwide (Top 15 cancer sites); **(b)** India (both sexes) (Top 10 cancer sites); **(c)** deaths in India males, females (Top 10 cancer sites) (Data Source: GLOBOCON 2022; International Agency for Research on Cancer).

Cancer is a foremost cause of death worldwide due to its high mortality rate. According to the International Agency for Research on Cancer (IARC), in the year 2022, mortality and incidence count were 9.7 million and 19.9 million, respectively. Number of new cancer cases are speculated to rise to 35 million in 2050 (2). The Indian population alone accounts for incidence and mortality rates of 1.4 million and 0.9 million, respectively, in 2022 (Figure 1a,b). Most of the deaths reported in India were from breast cancer and cervix uterine cancer in females. Whereas, cancers of the lip/oral cavity, lung, oesophagus and stomach topped the list in Indian males (Figure 1c). Oncology care is, therefore one of the fastest growing therapeutic fields. Early diagnosis and prognosis of a cancer type have become a necessity in oncology research, as it can facilitate the subsequent clinical management and prevention of death in cancer patients.

Cancer Burden: With more than one million cancers diagnosed every year in India, early identification and management are critical for developing a robust oncology care system in the country. This urgency is amplified by the vast population and the resultant burden on the healthcare infrastructure. However, population demand for healthcare services outstrips available resources in India, resulting in congested healthcare facilities, excessive waiting times, and often substandard services.

Rural-Urban Disparities: Rural communities frequently have insufficient healthcare facilities and poor infrastructure resulting in healthcare inequities across urban and rural populations. In the absence of proper logistics, people, particularly those in remote areas, are often deprived of quality healthcare services. For instance, the lack of transportation options and healthcare centres within a reasonable distance can lead to delayed diagnoses and treatments, significantly affecting patient outcomes.

Shortage of Healthcare Specialists: To meet the people's demands, an adequate number of healthcare specialists is also necessary. Yet, nurses, doctors, pathologists, specialized oncologists, and other medical personnel are in limited supply in India. Considering the number of allopathic doctors, the doctor-to-patient ratio is much lower than the World Health Organisation's (WHO) recommended guidelines of 1 per 1,000 population (3, 4). This scarcity can result in increasing workloads, fatigue among healthcare personnel, and a reduction in the quality of care, impacting the timely diagnosis and treatment of a significant number of patients.

### 1.2 How artificial intelligence can assist

Artificial Intelligence (AI) can assist India and other countries in overcoming healthcare challenges brought on by their large population. With AI and machine learning solutions, any large-scale cancer intervention may be successfully implemented, apart from making it more accurate and accessible. For instance, AI-driven diagnostic tools can analyse vast quantities of medical images and patient data to detect cancer at earlier stages with higher precision than traditional methods. These tools enable healthcare providers to identify subtle patterns and anomalies that might escape the human eye, significantly improving early detection rates. Similarly, AI-assisted surgery, which integrates robotics and machine learning, can enhance precision, reduce recovery times, and improve surgical outcomes.

AI also alleviates the burden on healthcare professionals by automating routine and repetitive tasks. Tasks such as patient data entry, appointment scheduling, and medication management can be streamlined through AI, allowing medical staff to focus more on patient care and less on administrative duties. Additionally, AI-powered monitoring tools can track patient conditions in real-time, alerting healthcare providers to critical changes, thereby enhancing patient safety and reducing the workload on medical teams.

AI-powered telemedicine platforms are particularly transformative in bridging the gap between patients and healthcare providers, especially in remote and underserved areas. These platforms enable patients to access medical consultations, follow-ups, and diagnostic services without the need for long-distance travel, ensuring that quality healthcare reaches even the most marginalized populations.

However, realizing AI's transformative potential in healthcare requires addressing several challenges. Key among these are the development of robust data infrastructure, the implementation of stringent data privacy regulations, and the establishment of secure, ethical data-sharing practices to protect patient confidentiality. Additionally, significant investments in technological infrastructure and the development of a skilled AI workforce are critical for effective implementation.

This review offers a comprehensive examination of AI's applications across various aspects of cancer management, including prevention, diagnosis, precision treatment, prognosis, and drug discovery. It also provides a detailed discussion on the roadmap and recommendations for integrating AI and telemedicine into India's oncology care landscape, while addressing the inherent challenges of AI adoption and highlighting recent advancements that tackle these critical issues. To refer to the detailed workflow methodology for article collection, please see Box 1.

Box 1 **Workflow methodology for article collection.**

The purpose of this review was to analyse the role and impact of artificial intelligence (AI) in the field of oncology. A comprehensive search was conducted using the PubMed academic database, employing search query with keywords such as “(((Artificial Intelligence) OR (Machine Learning)) AND ((Cancer) OR (Oncology)))” in the title or abstract of the articles published during last 10 years. Initially, 12,856 articles published had been identified. 41 more articles were included from citation search and other sources.

**Figure 3:**
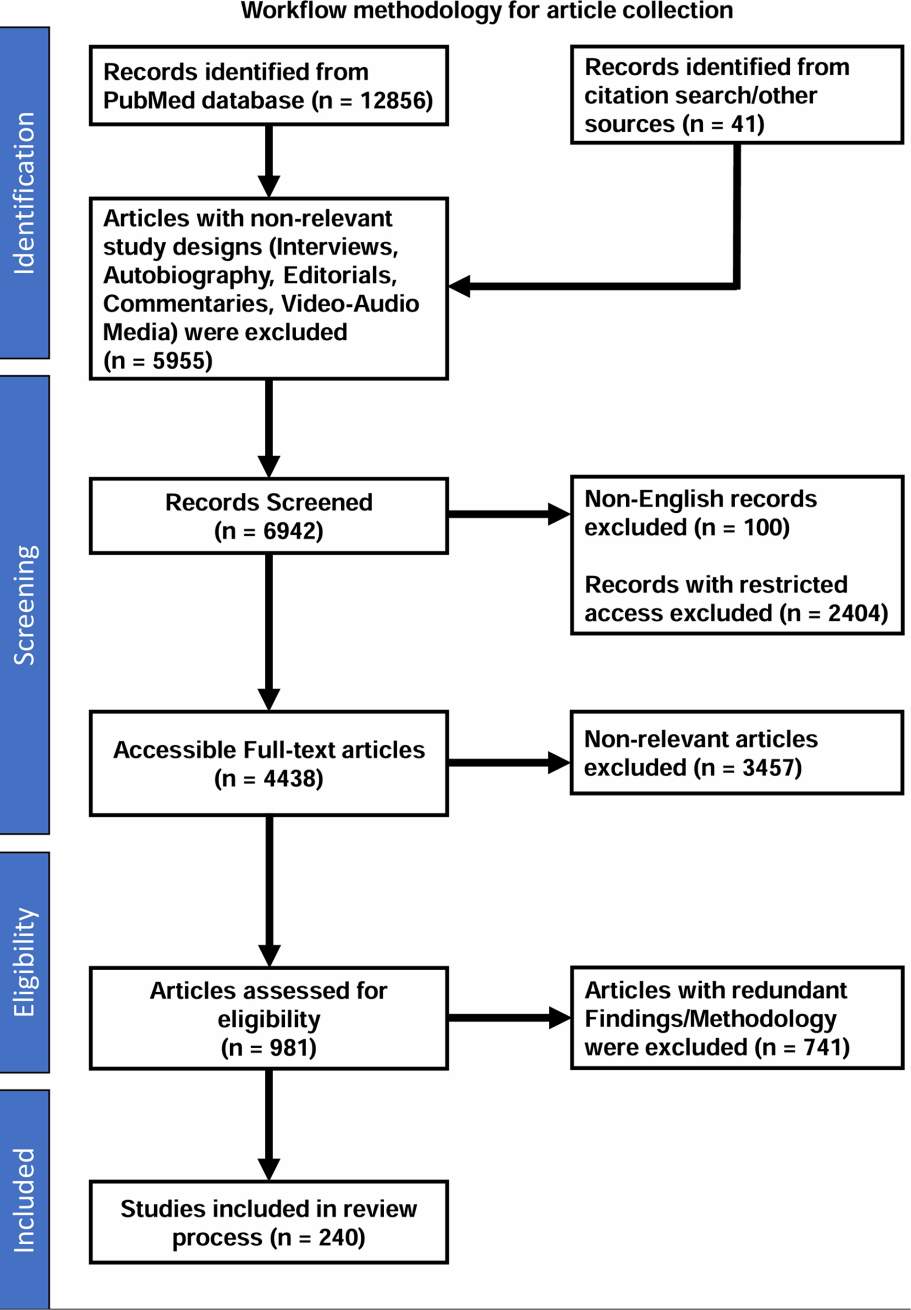
Workflow methodology for article collection. This workflow provides a detailed overview of the process used for the article collection in our review on the role and impact of AI in oncology. The workflow highlights the key stages, from identification and screening to eligibility assessment and final inclusion of relevant studies.

Articles with non-relevant study designs (Interviews, Autobiography, Editorials, Commentaries, Video-Audio Media) were excluded (*n* = 5,955). The collection was further refined based on four exclusion criteria, removing articles in the following categories: (i) non-English language (100 articles), (ii) Restricted access (2,404 articles), (iii) non-relevant articles (3,457 articles) and (iv) Redundant Findings/Applications/Methodology (741 articles). After applying these filters, 240 relevant articles remained, which were then analysed to understand the impact and role of AI in oncology (Figure 3).

### 1.3 What is artificial intelligence

**Figure 2:**
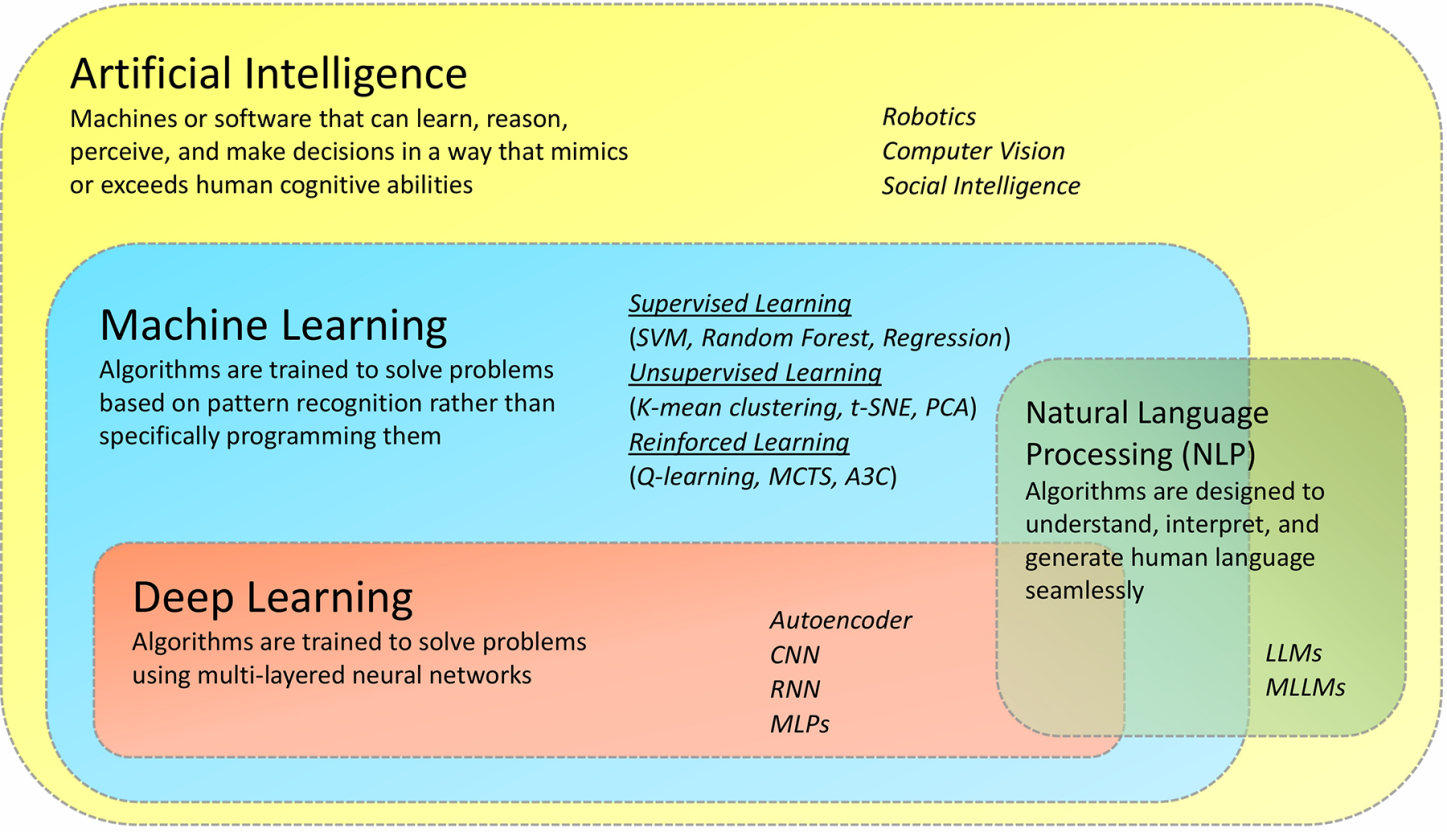
Artificial intelligence and its sub-disciplines. Although weak AI (ANI), and strong AI (AGI) are broadly defined based on their rigorous nature, there are other subfields of AI based on their applications. This includes- *machine learning*, which can be used to make predictions, classify data, and make decisions; *deep learning*, capable of learning complex patterns in data; *Natural Language Processing* for addressing the meaningful content of text-by-text miming and for translating languages) and *computer vision* (for images and video information) and *Robotics* for performing complex tasks.

AI is a field of research in which smarter algorithms are applied to mimic human intelligence (5). AI seeks to make it possible for machines to emulate and mimic human cognitive functions such as learning, reasoning, problem-solving, perception, and decision-making. Further, it can perform multitasking surpassing human abilities. It is a multifaceted field encompassing numerous specialized areas, such as (i) Natural Language Processing (NLP), it focuses on understanding and generating human language, covering applications like sentiment analysis and chat-bots (6); (ii) Computer Vision, it deals with visual data, enabling tasks such as object detection and facial recognition (7); (iii) Robotics, it combines hardware and software to create autonomous machines. Robotics has the potential to revolutionize industries like healthcare, transportation, and manufacturing (8); (iv) Social intelligence, is the ability of artificial systems to understand, interact with, and respond to humans in a socially and emotionally intelligent manner such as emotional recognition and feedback & learning (9). Machine learning (ML) is a sub-discipline of AI, that employs sophisticated algorithms on large-scale heterogeneous datasets to uncover valuable patterns that would otherwise be difficult or impossible to identify even for highly trained individuals (10). It is built upon three fundamental approaches or paradigms. Supervised Learning, the most common of these paradigms, involves training algorithms on labelled data to predict outcomes or make classifications on new, unseen data [popular models: Support Vector Machine (SVM), Random Forest and Regression]. On the other hand, unsupervised Learning operates without labelled data, instead uncovering hidden patterns and structures within datasets, frequently employed in clustering and dimensionality reduction tasks (popular models: K-means, t-SNE and PCA). Reinforcement Learning, the third paradigm, focuses on sequential decision-making, where an agent learns to interact with an environment to maximize cumulative rewards through trial and error [popular models: Q-learning, Monte Carlo Tree Search (MCTS) and Asynchronous Advantage Actor-Critic (A3C)] (11). Deep Learning (DL) is a subset of ML that uses neural network-based models to simulate the human brain's capacity for processing enormous amounts of complex data, including, but not limited to, image recognition, language processing, and drug discovery, all of which serve as a decision support system for researchers (Figure 2). Deep learning comprises a wide array of approaches and architectures that have propelled machine learning to new heights. Among these, Multilayer Perceptrons (MLPs) serve as the foundational building blocks, suitable for diverse tasks like regression and classification. Convolutional Neural Networks (CNNs) excel in processing grid-like data such as images, while Recurrent Neural Networks (RNNs) are designed for sequential data like time series and text. Transformers, with their self-attention mechanisms, have revolutionized natural language understanding. Autoencoders, another vital component, are employed for unsupervised learning and feature extraction, finding applications in dimensionality reduction and image denoising (12). Multimodal Large Language Models (MLLMs) and Large Language Models (LLMs) are part of both the Deep Learning and Natural Language Processing (NLP) domains. Large Language Models (LLMs) are designed to understand, generate, and analyse human language, significantly enhancing natural language processing tasks. MLLMs extend beyond LLMs by integrating multiple types of data, such as text, images, and audio, allowing them to handle multimodal information (13). These diverse deep-learning approaches are tailored to various data types and problem domains, marking them as the forefront of modern AI research and applications (Table 1). Since the human mind is only able to analyse a finite amount of data in a short duration of time, the exponential rise of AI over the last decade implies that it can serve as a sophisticated platform supporting human experts or clinicians to make the best possible decisions.

**Table 1:** Comparison of various AI models/algorithms with their applications in healthcare.

Algorithm/Model	Description	Applications in healthcare
Supervised learning		
Decision trees (CART, C4.5)	Decision trees are models that use a tree-like graph of decisions and their possible consequences. Each node represents a decision point, and the branches represent the outcomes. They are intuitive and easy to interpret.	Diagnostic decision support, predicting disease progression (14).
Random forests (RandomForestClassifier)	Random forests are ensembles of decision trees that aggregate the results of multiple trees to improve accuracy and control overfitting. They are robust against noise and have high predictive performance.	Patient risk assessment, predicting treatment outcomes (15).
Support vector machines (SVM)	SVMs are supervised learning models that find the hyperplane that best separates data into classes. They are particularly effective for high-dimensional data.	Disease classification, identifying biomarkers (16, 17).
Unsupervised learning		
K-means algorithm	K-means clustering is an algorithm that partitions data into k clusters, where each data point belongs to the cluster with the nearest mean. It is simple and efficient.	Patient segmentation, clustering symptoms for diagnosis (18).
DBSCAN algorithm	DBSCAN (Density-Based Spatial Clustering of Applications with Noise) is an algorithm that forms clusters based on density. It can find clusters of arbitrary shape.	Anomaly detection, identifying outliers in medical data (19, 20).
Principal component analysis (PCA) algorithm	PCA is a dimensionality reduction technique that transforms data into principal components, capturing the most variance with the fewest components.	Data visualization, noise reduction in medical data (21).
Reinforcement learning		
Q-Learning	Q-Learning is a reinforcement learning algorithm that learns the value of actions in states to achieve the highest cumulative reward.	Personalized treatment planning, optimizing clinical workflows (22).
Deep Q-network (DQN)	DQN combines Q-Learning with deep neural networks to handle high-dimensional data. It is effective in applications where complex decision-making is needed.	Robotic surgery, real-time treatment adaptation (23).
Proximal policy optimization (PPO)	PPO is a policy gradient method for reinforcement learning that balances exploration and exploitation. It provides stable training.	Adaptive therapies, dynamic treatment strategies (24).
Neural networks		
Convolutional neural network (CNN) (LeNet, AlexNet, VGG, Inception)	CNNs are specialized neural networks for processing grid-like data such as images. They consist of convolutional layers that extract features from images and are highly effective in medical image analysis.	Detecting tumors in radiology images (25).
Recurrent neural network (RNN) (LSTM, GRU)	RNNs are neural networks designed for sequential data. They use loops to maintain context over sequences and are used in healthcare for analysing patient records and predicting health outcomes over time.	Time-series health predictions (26).
Multilayer perceptron (MLP) (feedforward neural network, MLP classifier)	MLPs are classic neural networks with multiple layers of neurons. They are versatile and used in various healthcare applications.	Predicting disease outbreak, drug response prediction (27).
Autoencoder (variational, sparse autoencoder)	Autoencoders are neural networks that learn to encode data efficiently, reducing its dimensions and reconstructing it from the encoding.	They are used for detecting anomalies in patient data and compressing large datasets (28).
Natural language processing (NLP)		
Bidirectional encoder representations from transformers (BERT)	BERT is a transformer-based model that pre-trains on large text corpora to understand context.	It enhances clinical documentation and extracts insights from electronic health records (EHRs) (29).
Large language models (LLMs)		
Generative pre-trained transformer (GPT3,4)	GPT is a language model designed for text generation and understanding.	Text-based clinical decision support, medical literature review (30, 31).
Text-to-text transfer transformer (T5)	T5 converts all NLP problems into a text-to-text format.	Summarizing medical documents, translating medical texts (32).
Multimodal large language models (MLLMs)		
Contrastive language-image pre-training (CLIP)	CLIP integrates text and image data, allowing for advanced multimodal analysis.	Analysing medical images with textual reports, multimodal diagnostics (33).
Multi-modal vision and language model (VisualBERT)	VisualBERT combines visual and textual information, enabling tasks like visual question answering and generating image-based medical reports. It captures relationships between images and text effectively.	Visual question answering, generating image-based medical reports (34).
Unified transformer (UniT)	UniT processes multiple data modalities, making it versatile for complex healthcare tasks that require integrating diverse data types.	Multimodal data interpretation, combining patient imaging and clinical notes (35).
Robotics		
Robotics (Da Vinci surgical system, intuitive surgical)	The use of programmable machines to perform tasks autonomously or semi-autonomously.	Surgical assistance, patient rehabilitation, telemedicine, diagnostics, and stroke rehabilitation (36).

## 2 Artificial intelligence in oncology care

The AI approaches are already used widely in science and society for everything from clinical trials to robotics to self-driving cars. The power of machine learning has been demonstrated in healthcare by work on the human genome project, initiatives in cancer omics [such as The Cancer Genome Atlas (37), the International Cancer Genome Consortium, and the Clinical Proteomic Tumour Analysis Consortium], as well as numerous international machine learning competitions like the DREAM challenges (38, 39). When it comes to cancer detection, diagnosis, and treatment, the ability to gather and analyse sizable datasets on medical treatments and outcomes holds great promise for transforming medicine into a data-driven, outcome-oriented field. Early usages of machine learning in diagnosis and treatment have shown promise in detecting breast cancer from x-rays (40, 41), discovering new antibiotics (42), predicting gestational diabetes onset from electronic health records (43), and identifying clusters of patients who share a molecular signature of treatment response (44). AI-based algorithms hold great promise to pave the way to identify genetic mutations and aberrant protein interactions that can lead to disease diagnosis at a very early stage. AI-powered telemedicine and remote diagnosis can also aid in treatment planning and resource optimization for patients based on age, medical history, and genetic makeup. This personalized approach to disease treatment has the potential to improve patient outcomes. Government institutions as well as numerous other healthcare groups, find enormous potential in AI for enhancing cancer care.

**Figure 5:**
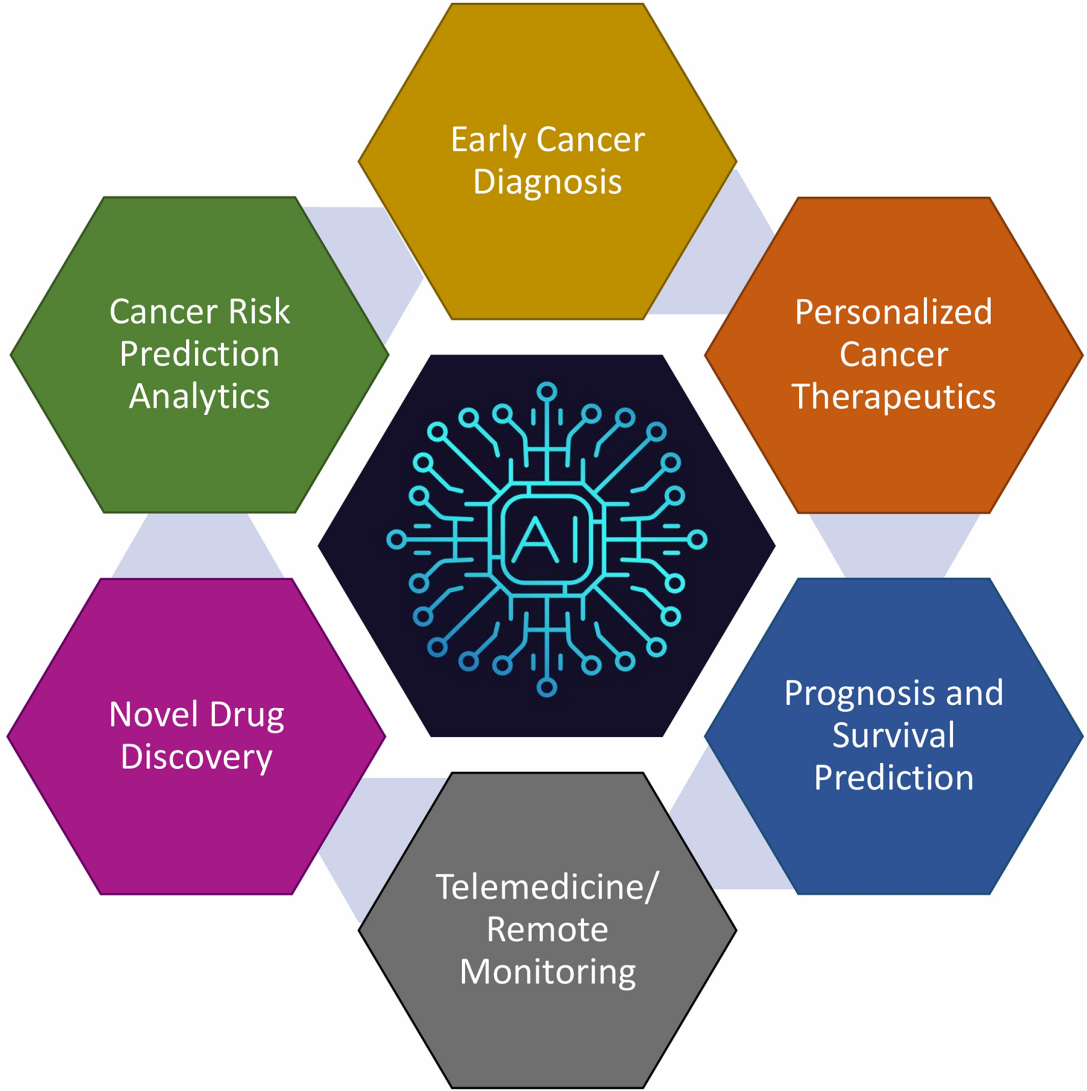
Key domains within oncology and research where artificial intelligence (AI) can significantly impact patient care and scientific advancement. While each of these domains may appear disjoint, they are in fact seamlessly integrated with AI playing a significant role in this process. AI-based tools can screen data from an enormous volume of subjects to find patterns and connections between them. This information can not only assist in personalized cancer care but can also recommend the most appropriate therapy as well as digital communications for networking a large number of centres.

In the context of oncology, with the advancement of technology more and more clinical data is being generated every day, and AI has become essential for the automated analysis of massive patient data, such as biomedical images, genetic profiles, health reports, molecular tests, etc. to detect abnormalities/patterns and forecast the possibility of cancer or response to treatment. AI can improve the efficiency and accuracy of personalized risk assessment, early diagnosis, staging, therapeutics, clinical decision-making, prognosis, and survival predictions (Figure 5). In India, institutions like the All-India Institute of Medical Sciences (AIIMS) and various state-run healthcare facilities are exploring AI's potential to enhance cancer care. We provide here an overview of some major areas of oncology and research where AI can have substantial influence (Table 2).

**Table 2:** Applications of AI in oncology care.

Applications of AI	Description	Benefits
1. Predictive analytics	Accurate prediction of cancer occurrence in high-risk individuals and timely identification of asymptomatic cancer patients utilizing AI-based algorithms trained on diverse datasets, including: i). Medical imaging (Breast Mammograms, Chest x-Rays), ii). Large-scale genomic datasets (Cancer-related mutations/variants, metabolic biomarkers, signatures of DNA methylation, expression profiles of cancer-related genes or non-coding RNAs), iii). Comprehensive electronic health records data (Patients’ medical history, family history, demographics, test reports)	Enables early intervention, potentially reducing cancer incidence. Improves patient outcomes and survival rates through timely treatment.
2. Cancer diagnosis and staging	Accurate diagnosis of cancer and classification into Grade/Stage & Primary Site/Histological Type utilizing AI-driven platforms that can integrate multimodal datasets such as: i). Radiological Images (CT, x-rays, MRI, PET scans, and ultrasound), ii). Videos (such as colonoscopy for malignant polyps), iii). Fundus images for the identification of retinoblastomas, iv). Digitized H&E-stained slides from core needle biopsies, v). Dermoscopy images for Skin Cancer, vi). Large-scale genomic data (mutational signatures, gene expression, DNA methylation, miRNA profiles), vii) Peripheral blood biomarkers (protein biomarkers, circulating cell-free tumour DNA) from liquid biopsies	Enhances early detection, leading to better treatment outcomes. Reduces diagnostic errors and enhances accuracy.
3. Treatment and response assessment	Personalized Treatment: Clinical Decision Support System (CDSS) for data-driven personalized cancer therapy recommendations, integrating automated radiation therapy plans based on radiological data, genomic information, and medical records. AI-Assisted Surgery: Enhanced preoperative surgical planning, intraoperative navigation with real-time feedback using AR/VR technologies, and postoperative monitoring using data feeds from monitoring devices. AI-programmed robotic surgery for increased accuracy and minimizing human error. Prognosis/Survival Prediction: Enhancing precision in forecasts for chemoresistance, cancer recurrence, and survival outcomes using multimodal AI algorithms that can integrates multi-omics data and other complex multi-dimensional datasets.	Optimizes treatment plans for individual patients. Enhances treatment efficacy and minimizes adverse effects. Improves overall patient care and satisfaction.
4. Telemedicine/Remote monitoring	Apps/Websites: AI-powered chatbots and virtual assistants for preliminary assessments, symptom tracking, diagnosis, connecting with a specialised doctor (telemedicine), tele-education, basic medical advice, medication reminders, community support. Wearable Devices: AI-powered real-time remote monitoring of behavioural patterns (Sleeping habits, physical activity, heart rate, stress levels) and Cell phone images for remote patient monitoring, cancer risk prediction, ultra-personalized diagnosis and treatment recommendations.	Improved access to healthcare services, especially in remote or underserved areas. Reduces the need for in-person visits and hospital readmissions.
5. Novel discovery	Drug Targets/Biomarkers: AI-driven prediction of therapeutic targets in oncology utilizing population-specific publicly available mutation/loss-of-function screen profiles, gene expression datasets, and clinical data. Drug Designing: AI-powered tools like AlphaFold, DeepNeuralNetQSAR, DeepChem, DeepTox, and gene2drug for the prediction and identification of drug molecules with enhanced biochemical properties for improved sensitivity and specificity, alongside a reduced risk of adverse effects. Additionally, optimisation of existing drugs with enhanced biochemical properties for better performance. Clinical Trial: Enhanced patient eligibility decisions, automated patient recruitment processes, optimized trial design, and real-time monitoring of patient adherence to protocols for significantly improved overall efficiency and effectiveness of cancer clinical trials.	Speeds up the drug discovery process, leading to innovative treatments. Reduces research and development costs and time with improved success rates.

### 2.1 AI for cancer risk predictive analytics and early intervention

The first step in managing cancer is to prevent it, and the key to that is to identify those who are most at risk of contracting the disease. A quick and early mass screening is required for a population to address its health priorities. As a result, automated AI-based methodologies are now required over manual ones like breast examinations, Pap Smears, HPV tests or visual inspections. Incorporation of AI-based programs in routine healthcare can transform cancer care in India by identifying high-risk asymptomatic individuals so that timely preventative policies like vaccinations and lifestyle modifications can be devised and recommended.

Researchers globally, including those in India, have successfully developed AI-based programs to analyse diverse forms of biomedical imaging data for the early prediction of cancer. An AI-based algorithm was implemented (53), in a study published in the Journal of the National Cancer Institute to forecast the risk of breast cancer based on mammograms and clinical data. According to Rodriguez-Ruiz et al., the algorithm had a 70.4% accuracy rate in predicting women at a high risk for developing breast cancer within 5 years. In a recently published study, it was concluded that integrating 5 different AI-based deep learning algorithms outperformed cancer risk as predicted by the Breast Cancer Surveillance Consortium (BCSC). This was further enhanced by combining AI and BCSC models (54). Specifically in Breast Cancer screening, tools like ScreenPoint Medical's Transpara, CureMetrix's cmAssist, Qlarity Imaging's QuantX AI algorithms and BUCAD claim to support the identification of cancer-specific indicators through mammography, MRI or ultrasound images for improved accuracy and efficiency of early detection (55–58). Various other studies have also assessed the capability of mammography-trained AI systems to show significant gains in risk prediction compared to clinical risk models alone (59, 60). For a detailed overview of the AI-workflow for image-based classification of breast mammograms, please refer to Box 2. A deep learning-based CNN model could enhance screening by forecasting the likelihood of lung cancer among asymptomatic smokers using chest x-rays and limited EHR data (61). An individual's predisposition to developing certain cancers can also be predicted using AI by analysing large-scale genomic datasets (62). Google created a deep learning-based AI algorithm called DeepVariant to discover genomic variants from sequencing data. It has been used in cancer genomics to accurately identify cancer-related mutations and forecast the likelihood of developing the disease. Breast and lung cancer are just two cancer types where the algorithm has demonstrated promising results (63). In one of their research, Listgarten et al. created an SVM machine learning model to utilize single nucleotide polymorphisms (SNPs) profiles of steroid metabolizing enzymes (CYP450s) to precisely predict the development of “spontaneous” breast cancer. AI-generated Polygenic Risk Scores (PRS) to evaluate a person's chance of acquiring certain cancer has been developed using genetic data from numerous variants. PRS has been successfully employed in the assessment of multiple cancers and is shown to support individualized risk assessment by categorizing people into several risk groups (64–66). Other than genomic variants, metabolic biomarkers, signatures of DNA methylation and expression profiles of cancer-related genes or non-coding RNAs can predict an individual's predisposition to certain cancers or their response to a treatment regime. Employing machine learning strategies to forecast the likelihood of acquiring cancer based on the above metrics is increasingly becoming common in oncology care (67–72). AI can also find patterns and relationships associated with cancer risk using comprehensive electronic health records data. Patients' medical history, family history, medication history, demographics, age, gender, clinical notes, test reports, symptoms, and treatment outcomes can all be fed into an AI model to predict the likelihood of developing cancer. Automated models to predict breast and lung cancer (42, 73–75), using a combination of the above-mentioned clinical records are already being developed and successfully implemented.

Box 2 **AI-workflow for image-based classification of breast mammograms.**

**Figure 4:**
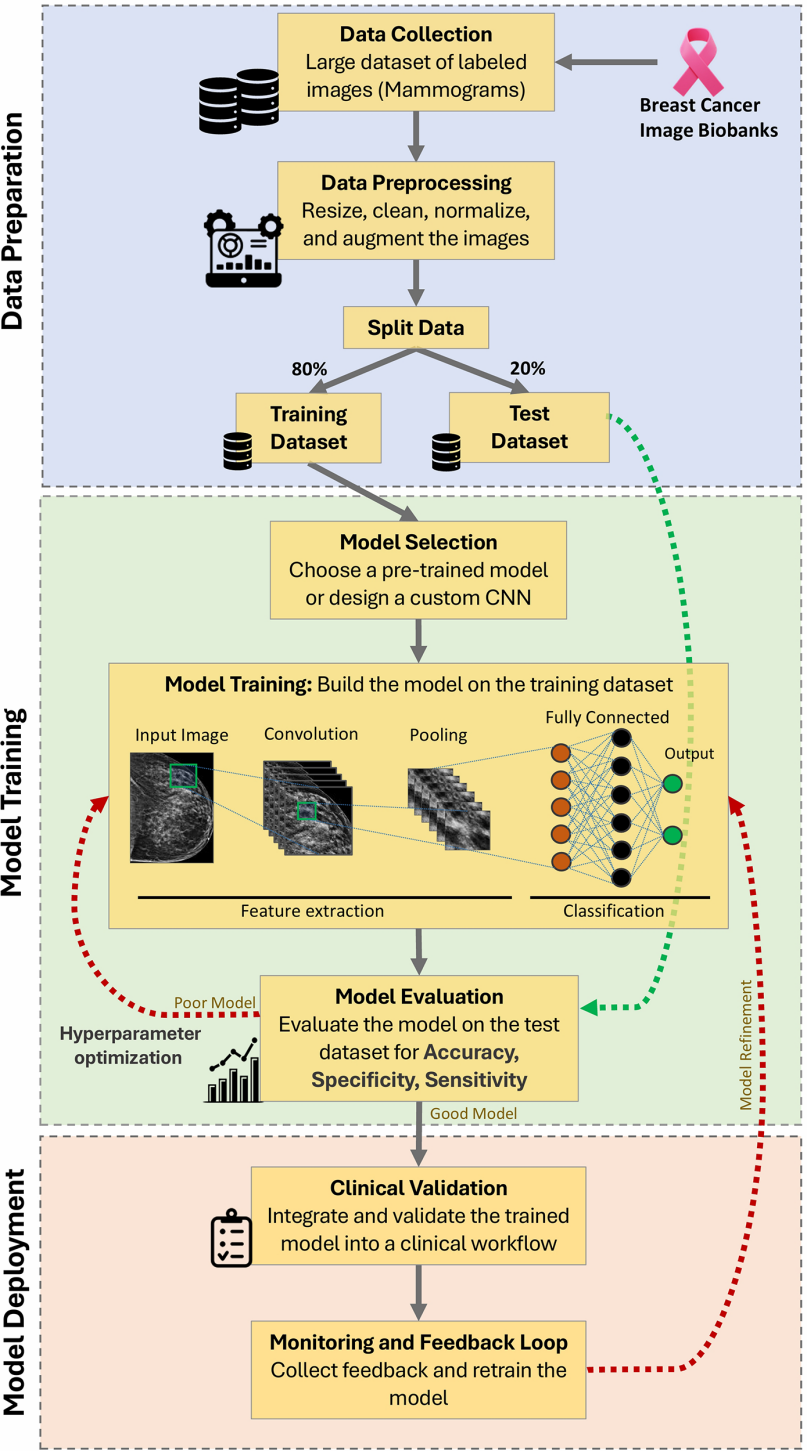
AI workflow for image-based classification of breast mammograms (240). This schematic illustrates the step-by-step workflow of an AI system using a Convolutional Neural Network (CNN) algorithm. Key stages include data collection, preprocessing, training/testing split, model selection, feature extraction, classification, evaluation, hyperparameter optimization, clinical validation, and continuous monitoring for improved accuracy.

The basic workflow begins with data collection from breast cancer biobanks, ensuring a diverse and comprehensive dataset, such as The Cancer Imaging Archive (TCIA) (45). Next, data preprocessing steps, including normalization using tools like ImageJ (46) and augmentation methods such as rotation, scaling, and flipping via libraries like TensorFlow and Keras, are performed to enhance the quality and variability of the data (47, 48). The dataset is then split into training (80%) and test datasets (20%) using methods such as stratified sampling to enable robust model validation (49). During model selection, various architectures are considered, with a focus on convolutional neural networks (CNNs) like VGG16x (50) or ResNet (51) due to their proficiency in image analysis. Model training involves feature extraction through convolutions and pooling layers, using frameworks like PyTorch (52) or TensorFlow (47), followed by classification. The model outputs a probability score indicating the likelihood of malignancy, which is used to categorize the mammogram as benign, malignant, or requiring further review. The trained model is evaluated against the test set using metrics such as accuracy, sensitivity, specificity, and AUC-ROC curves (49). If the performance is suboptimal, hyperparameter optimization techniques, such as grid search or random search via tools like Scikit-Learn, are applied. Conversely, a well-performing model undergoes clinical validation with real-world datasets to ensure its reliability. Finally, a monitoring and feedback loop is established to continuously assess and refine the model, incorporating new data and improving accuracy over time through continuous integration/continuous deployment (Figure 4).

Given India's resource limitations and cultural challenges, efforts are underway to create affordable and accessible cancer screening solutions (76). The Indian MegCan Care project, a collaborative AI-driven initiative by the Meghalaya government, Apollo Telemedicine Networking Foundation, and the World Economic Forum, seeks to provide free cancer screening to one million individuals. It focuses on early detection of oral, breast, cervical, lung, and esophageal cancers, aiming to enhance timely diagnosis and patient outcomes through widespread screening programs (77). Innovations like Thermalytix, a radiation-free AI tool using thermal imaging, demonstrate significant promise for population-level breast cancer screening in resource-constrained settings, boasting a positive predictive value of 81% (78).

### 2.2 AI for early cancer diagnosis and staging

Cancer is a heterogeneous disease harbouring a spectrum of genomic and epigenomic alterations with large variations in tumour fitness, mutational burden and responsiveness to conventional chemotherapy, and biological behaviours (both within and between cancer types). Numerous cancer forms may also exhibit comparable clinical signs or share similar symptoms along with the biomarkers employed to identify and categorize cancers, thus causing uncertainty in the diagnosis and staging of cancer. Further, due to insufficient knowledge or lack of information, diagnosing rare cancers makes interpretation even more difficult. Additionally, because of differences in how pathologists or radiologists interpret genetic tests, histopathology slides, and imaging results, inter-observer variability affects the consistency and accuracy of cancer diagnosis and staging. The incorporation of AI can enhance the speed, accuracy, and consistency of result interpretation from radiological images, digitized pathology slides, and colonoscopy procedure footage, as well as pictures of skin lesions, to determine the presence of cancer, the subtype categorization, the grade, and the purity of the tumour. AI can assist in mass screening of symptomatic patients for early cancer detection, galvanizing towards personalized treatment regimens by overcoming the shortage of healthcare professionals and improving accessibility to timely diagnosis.

A study demonstrated by McKinney and others (41) proved the utility of AI in the successful screening of breast cancer using mammograms. A group at Google designed a CNN-based screening model for identifying and classifying abnormal nodules from CT scans for lung cancer diagnosis with more accuracy and efficiency (79). Similarly, x-rays, MRI scans, PET scans, and ultrasound images have also been utilized to improve diagnosis, characterization as well as prediction of metastasis in several cancer types using machine learning approaches (80–83). AI algorithms have also been designed to accurately detect and categorize malignant polyps from clinical colonoscopies in real-time with high sensitivity and specificity (84, 85), and skin cancer from dermoscopy images with a level of competency comparable to dermatologists (86). Additionally, an AI algorithm based on deep learning models like CNNs and RNNs can identify the presence of cancer, grade, and assess tumour purity estimates from digitized H&E-stained slides of prostate tissue from core needle biopsies (87); classify cancer type and access invasiveness based on breast biopsy images (88–91); identify retinoblastomas from fundus images with high specificity (92). Artificial intelligence (AI) algorithms can also learn to recognize molecular markers suggestive of cancer by comparing genomic patterns between diseased and non-cancerous tissues. These models can subsequently use the genetic profiles of new samples to categorize them, aiding in the correct detection of cancer (93–95). AI algorithms have been used to analyse large-scale genomic data, including mutational signatures (96), gene expression (97–99) DNA methylation (100, 101), and miRNA profiles (102). In 2006, NIH/NCI launched a landmark cancer genomics program “The Cancer Genome Atlas (TCGA) Research Network” with the goal of gathering, profiling, and analysing a sizable number of human clinical tumours representing various tumour types and their subtypes to identify molecular aberrations at the DNA, RNA, protein, and epigenetic levels. Integrative analysis of this data using machine learning has made it possible to identify molecular patterns, common pathways, master regulatory hubs that are activated or deactivated across many tissue types, and molecular phenotypes and biomarkers particular to different cancer stages (103–107). Using hundreds of somatic mutation data, the Pan-Cancer Analysis of Whole Genomes (PCAWG) Consortium utilized a DL model to forecast the origins of 24 cancer types both individually and collectively (108, 109). Automated screening and staging models based on NLP (AI-based) have also been developed to analyse unstructured data from clinical reports for the identification of cancer-specific abnormalities (110–112). Analysis of liquid biopsies for the identification of peripheral blood biomarkers of diagnostic relevance has recently gained interest (113–117). Integrating artificial intelligence with Raman spectroscopy, a group designed a highly accurate DL model to analyse liquid biopsies for lung cancer diagnosis (118). The CancerSEEK model is another ground breaking example of the utilization of AI in the early diagnosis of multiple cancers based on the presence of specific genetic mutations in circulating cell-free tumour DNA and the levels of protein biomarkers using immunoassays from liquid biopsies. It demonstrated high predictive value for the number of common cancer types; especially for ovarian and liver cancer (119).

From India, iOncology.ai is a cutting-edge AI-driven platform, developed by the All-India Institute of Medical Sciences (AIIMS), New Delhi, in collaboration with the Centre for Development of Advanced Computing (CDAC), Pune, under the auspices of the Ministry of Electronics & Information Technology. Launched in 2024, the platform leverages deep learning algorithms to analyse radiological and histopathological images with high precision, facilitating early detection and personalized treatment of breast and ovarian cancers. The AI model is trained on an extensive dataset comprising approximately 500,000 images from 1,500 patient cases and is currently undergoing validation in district hospitals across India (120). In another significant initiative, the Apollo Cancer/Radiology Centre, in partnership with Google Health, is developing an AI-driven system for the early diagnosis of cancer using imaging data. This collaboration aims to harness AI technology to improve the accuracy and efficiency of cancer detection, addressing the shortage of radiologists and offering specialized screenings to enhance healthcare outcomes (121).

### 2.3 AI in cancer treatment and response assessment

Following a cancer diagnosis, the treatment outcome varies depending on the individual's genetic make-up and aggressiveness of the tumour. However, the dose and the choice of intervention (neoadjuvant, surgery, chemotherapy, radiotherapy, immunotherapy, etc.) are still empirical without a solid scientific basis. AI can assist clinicians in designing personalized and appropriate treatment plans, monitoring response to the treatment, and in predicting recurrence risk and patient survival, thus lowering healthcare costs, and workload and improving the efficiency of cancer management.

#### 2.3.1 Personalized treatment

AI can successfully evaluate complicated datasets to predict cancer prognosis, thereby assisting physicians in making better-informed treatment decisions and enhancing patient care. By analysing a patient's genetic information, omics data, medical history, and treatment response data, AI algorithms can uncover novel biomarkers linked to drug response and can predict which treatments will be most effective for an individual patient. This approach of precision medicine can improve the efficacy of cancer treatments while reducing the risk of adverse drug-associated side effects. Oncologists can choose the most efficient and individualized treatment options for their patients by utilizing the power of AI to calibrate drugs and increase their pharmacological action efficacy (122–126). IBM Watson for Oncology is a well-known instance of an AI-based treatment recommendation system created by IBM in partnership with Memorial Sloan Kettering Cancer Centre to help oncologists make data-driven treatment decisions for cancer care. This platform examines a patient's medical records, including clinical notes, pathology results, and molecular profiling data, and compares this information with a sizable collection of medical literature, trial data from clinical studies, and professional therapy recommendations. Watson for Oncology develops tailored, real-time treatment recommendations based on this research and predicts disease prognosis, highlighting prospective therapeutic choices (127–129). Another deep learning-based Clinical Decision Support System (CDSS) was created by Printz et al. in 2017, and it is capable of extracting and analysing a significant quantity of clinical data from medical records and generating cancer therapy alternatives highlighting the value of AI technology in aiding physicians in enhancing cancer patient treatment regimens (130). A precision oncology knowledge repository called OncoKB is another example of an AI-based platform that uses clinical data and genomic information, providing details on the therapeutic ramifications of certain mutations. Identifying targeted treatments, forecasting cancer risk, and gauging therapy efficacy are all made easier with the help of OncoKB (131). Based on genetic variants and clinical parameters, AI models can forecast a particular individual's susceptibility to chemotherapy-related toxicity, helping oncologists make informed decisions about dosages and combined treatment plans to improve efficacy and tolerance (132). Dorman et al. developed a machine-learning algorithm to forecast how well breast cancer will respond to chemotherapy. It was able to distinguish between the effects of two chemotherapy medications, taxol or gemcitabine, by investigating the association between chemotherapy treatments and patients' genetic profiles (133). Also, an AI-based platform was developed that could accurately evaluate the effectiveness of immunotherapy in PD-1-sensitive patient with advanced solid tumours (134, 135). Utilizing the HLA mass-spectroscopy database, an AI model could help identify cancer neoantigens more accurately and boost the effectiveness of cancer immunotherapy (136). Machine learning algorithms have also been utilized to associate radiomic biomarkers (quantifiable features like texture patterns, structural changes, or intensity variations) within the biomedical images (CTs/MRIs) of tumour regions with treatment response and survival, thus, helping oncologists in anticipating favourable responses to therapies (137–140). AI algorithms can aid in radiation therapy planning by segmenting tumours and organs at risk from medical imaging scans. AI-based contouring algorithms have been created to automate the delineation of organs and tumour volumes in CT and MRI images, potentially assisting radiologists in mapping out treatment targets or autonomously planning radiation schedules (141, 142). Utilising deep-learning technology, the software can now generate automated radiation therapy plans within a matter of a few hours without human intervention (129).

#### 2.3.2 AI-assisted surgery

Using imaging data, AI can help with preoperative planning by giving surgeons accurate 3D representations of the patient's anatomy. This enables surgeons to see the tumour and surrounding tissues more, allowing for more accurate surgical planning. 3D images based on CT scans and MRIs have been utilized to predict the pathological grade of hepatocellular and brain carcinoma for better preoperative planning and reduced complications (143–147). AI can also help with intraoperative navigation by giving the surgeon feedback in real-time while the surgery is being done by overlaying 3D pictures on the surgical field using augmented reality (AR) or virtual reality (VR) technologies. As an illustration, a 2020 study that was published in Nature Medicine employed augmented reality to direct the excision of brain tumours based on real-time stimulated Raman histology images, increasing surgical accuracy (148). One of the newest advancements in minimally invasive surgery is robotic surgery. Artificial intelligence (AI) algorithms can be programmed into robotic surgery systems to identify and avoid harm to healthy tissues during surgery, lowering the chance of inaccuracies and collateral damages (149–151). Researchers from the Children's National Health System in Washington, D.C., developed the SMART system, which is intended to autonomously carry out soft tissue procedures, including cancer surgeries. It uses AI algorithms and image processing to direct the robotic arm in real-time while the procedure is being performed, increasing accuracy and minimizing human error (152). AI can also help with postoperative monitoring by evaluating multiple data feeds from sensors and other monitoring equipment such as blood pressure monitors or ECGs to identify early indicators of complications, enabling quick intervention and treatment (153).

#### 2.3.3 Prognosis/survival

Cancer prognosis entails predicting disease recurrence, residual cancer burden, metastasis, patient's overall survival, and progression-free survival with the goal of improving patient care. Utilizing integrated multi-omics data (154–156) and other complex multi-dimensional datasets (157, 158), ML techniques have been proven to increase the precision of forecasts for cancer recurrence, and survival outcomes and aid doctors in the precise estimation of prognosis and treatment plan customization both pre and (159–162) post-intervention (163–165) scenarios, taking into account elements including tumour size, lymph node involvement, and hormone receptor status. A group in China has developed an algorithm that could analyse patient chemoresistance and prognosis and accurately predict the recurrence risk of breast cancer patients from EHR data (166). These approaches can assist doctors in developing customized treatment plans and follow-up care (167).

### 2.4 AI in telemedicine/remote monitoring

Post-COVID-19 pandemic, the healthcare sector has significantly changed and has sped up the global deployment of telemedicine tools and telehealth services. Telemedicine has the potential to bring about transformative changes in cancer care in India. The practice of providing cancer care remotely through video, telephone, and other electronic communication methods is known as Teleoncology or telemedicine in oncology. Teleoncology applications include improved access to specialized care, cancer clinical trials, cancer telegenetics, telepathology, preoperative counselling, remote chemotherapy supervision, symptom management, follow-ups, survivorship care, and palliative care for people who might live in distant or underserved locations or have limited access to medical facilities support (168).

#### 2.4.1 Apps/websites

AI-powered chatbots and virtual assistants can conduct preliminary assessments, diagnose symptoms, and offer basic medical advice. They can aid in classifying patients according to the seriousness of their conditions, allowing medical personnel to set priorities and distribute resources appropriately, thus facilitating effective healthcare delivery. One AI-based hospital pharmacy service was successfully tested in China during the COVID-19 pandemic in 2020 (169, 170). Hopido is an Indian Startup that has developed Cancer Dost, an AI-enabled chatbot to assist patients in connecting with specialized doctors and provides guidance for cancer treatment (171). As a tool for home-based treatment, mobile applications improve symptom management, lifestyle adjustment, and medication adherence. The oncologist can benefit from telemedicine's interactive tele-educational resources. AI-based algorithms for skin cancer diagnosis have recently been included in a number of mHealth apps, making this method available to the general public, allowing laypeople to use an AI-based mHealth app to assess their condition and determine if a doctor visit is necessary (172). An AI-powered app called Cancer Aid was created to help cancer patients along their journey. It gives access to a community of other cancer patients for support and direction, as well as individualized treatment information, symptom tracking, medication reminders, and access to symptom tracking (173). Using AI technology, CancerBASE offers individualized nutrition advice to cancer patients based on their individual medical and genetic profiles. To promote treatment outcomes and general well-being, it attempts to optimize nutrition (174).

#### 2.4.2 Wearables/devices

With commercialization and the growing use of wearable technology, mobile health apps, and social media posts, AI can also make use of information on a variety of lifestyle and behavioural patterns, including risky behaviour, physical activity, sound sleep, alcohol consumption, smoking, and stress levels. Integrating these data into AI algorithms can revolutionize cancer care by improving cancer risk prediction and patient monitoring (175–177). Healthcare professionals may monitor the health of cancer patients without having to make regular in-person visits thanks to AI-powered remote monitoring technologies. For instance, wearable technology powered by AI algorithms can continuously track vital signs, spot anomalies, and notify medical staff when intervention is required. Innovative wearable technology makes it possible to track and analyse each patient's data differently based on their physical and genetic risk factors, resulting in a highly ultra-personalized diagnosis and course of treatment and better patient outcomes. This enables faster treatment and reduces the burden on medical facilities. This facilitates prompt treatment and lightens the load on medical institutions (178, 179). Internationally, more than 70 AI-based devices have already been approved by the FDA with at least 50% of them being related to cancer radiology, clinical oncology, and pathology (180). One well-known example is the handheld device iBreastExam (iBE) (UE Life Sciences, Philadelphia, PA), which was created to do breast examinations for suspected anomalies or lumps in the breast tissue in a painless, non-invasive manner (181, 182). In a study done on women from the Nigerian population, this portable equipment revealed superior sensitivity (63%) for the identification of breast abnormalities as compared to clinical breast examination (CBE) (183). This radiation-free device can be particularly beneficial for early breast cancer detection campaigns in underserved communities or in regions with limited access to state-of-the-art healthcare facilities with only a little training. With increasing subscribers nationwide, surveillance using mobile phones integrated with machine learning programs has the potential to extend the reach of clinicians and vital diagnostic care to remote areas in India. A further possibility to enhance cancer screening may be the use of mobile devices to examine skin lesions. Cell phone-based triage or monitoring are prospective future methods for more efficiently deciding which patients to refer to clinicians or for expanding care in resource-limited areas, even though making this technology clinically feasible is still a work in progress.

### 2.5 AI in novel discoveries

**Figure 6:**
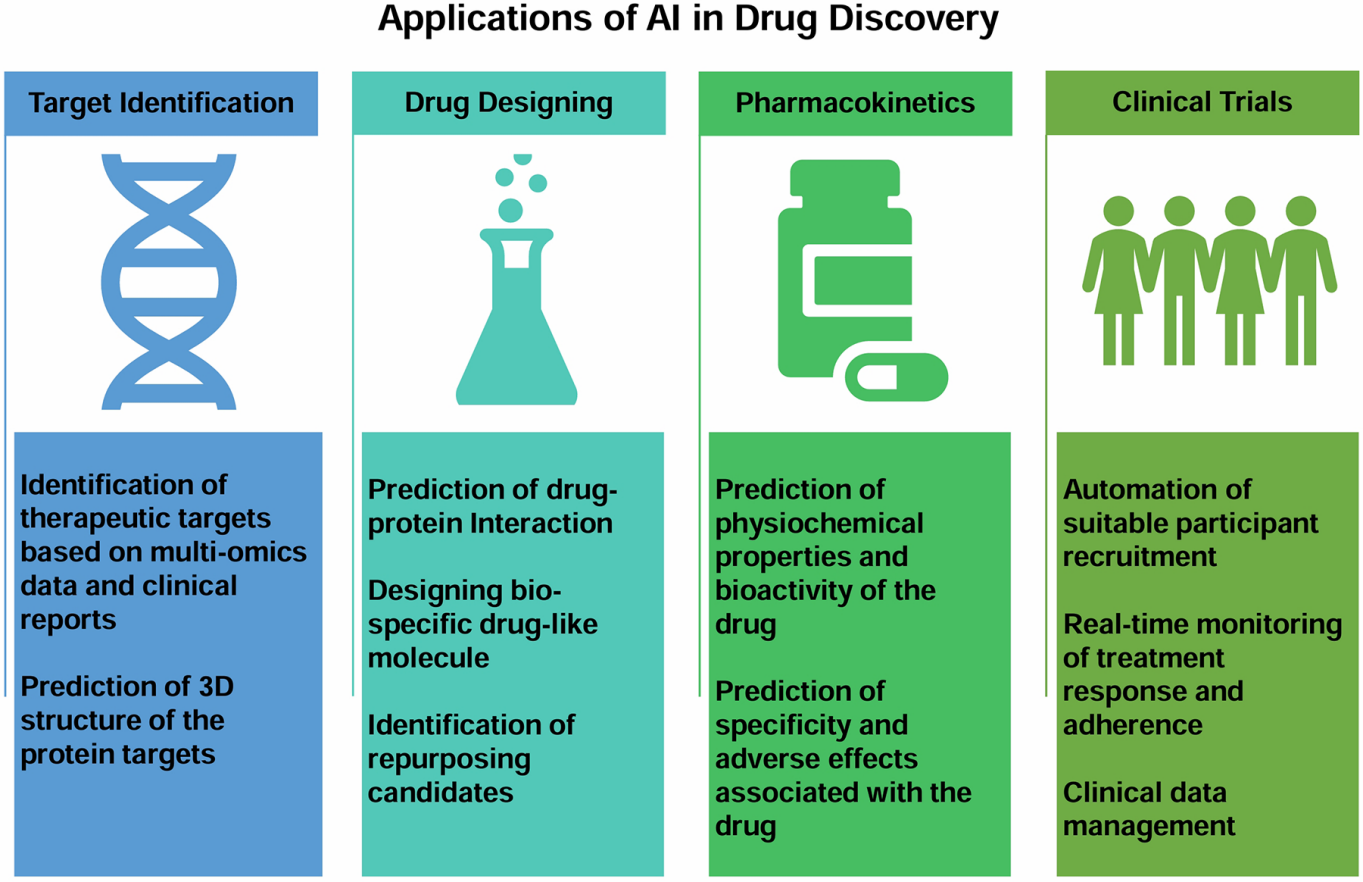
Figure showing broad applications of AI in the areas of novel drug discovery from target identification, drug designing, pharmacokinetics to clinical trials.

India has one of the most genetically diverse populations in the world, providing researchers with a unique chance to look into the molecular roots of diseases and create specialized treatment strategies. Additionally, the high prevalence of cancer enables researchers to investigate many subtypes, uncover the natural history and molecular basis of these diseases, and find possible targets for cutting-edge treatments. The growing availability of huge cancer datasets (public or private) and affordable access to various NGS and imaging technologies have sparked an explosion in interest in using AI to speed up discovering novel drugs/targets, which is frequently time and financially expensive. AI methods have been used in several areas of cancer research, including digging into molecular basis, creating anti-cancer drugs, and carrying out randomized controlled trials (RCTs) (Figure 6).

#### 2.5.1 Drug targets/biomarkers

For example, a support vector machine model was used in a study to derive features that might predict possible therapeutic targets in liver cancer using clinical data, gene expression patterns, and protein-protein interaction networks (184). Another group developed a deep learning-based categorization strategy to identify proteins linked to the pathogenesis of breast cancer and report promising candidates for biomarkers or drug targets (185). By utilizing both gene-specific and cell-line-specific data, the ECLIPSE machine-learning technique could predict cancer-specific therapeutic targets based on the publicly available loss-of-function screen datasets from the DepMap consortium (186, 187). Mkrtchyan et al. used the artificial intelligence-powered target discovery platform PandaOmics to investigate alterations in gene expression in rare DNA repair-deficient disorders and found new cancer targets (188). Utilizing the TCGA data, the ML approach established the role of F-box/WD repeat-containing protein 7 (Fbw7) in cancer cell oxidative metabolism (189).

#### 2.5.2 Drug designing

AI has also been used to design drugs with desired physiochemical properties and target specificities. Using an AI-powered protein structure database called AlphaFold researchers from the University of Toronto and In-silico Medicine developed a drug that could treat hepatocellular carcinoma (HCC), or liver cancer (190). Instead of the typical length of about a year, a research group developed a DL model and identified potent inhibitors of the discoidin domain receptor 1 (DDR1), a kinase target implicated in several malignancies (191). Another study used the ML approach to improve the sensitivity and specificity of dual inhibitor cyclin-dependent kinases four and human epidermal growth factor receptor 2 (192). AI approaches are also deployed to predict adverse effects linked to drug toxicity and to design molecules with better biochemical properties in terms of solubility, synthesizability and drug-likeness (193–195). Various AI-based computer tools have been developed that can help identify cancer-related drugs like DeepNeuralNetQSAR, DeepChem, DeepTox, gene2drug, STITCH, etc. (196). The identification of repurposing candidates from medications that can reverse the expression profiles of cancer-specific gene signatures has also been done using transcriptional data sets from LINCS (Library of Integrated Network-Based Cellular Signatures) libraries and other sources (197).

#### 2.5.3 Clinical trial

Adoption of novel cancer therapies depends on the success of clinical trials, and finding the right subjects to enrol or recruit is seen to be the most challenging aspect of it. This requires labour-intensive work to match potential subjects with eligibility requirements satisfying inclusion and exclusion criteria. AI can improve cancer clinical trials by locating suitable participants for clinical trials and in the design of trials that are more effective and efficient (198). For instance, to automate the patient recruitment process, the combination of multi-layer perceptron modelling and natural language processing was used to extract pertinent data from patient records to help compile evidence for better patient eligibility decisions (199, 200). Analysis of the clinical trial data can assist in speeding up the discovery of new drugs and help patients receive new cancer treatments more rapidly by identifying patients who are most likely to benefit from new therapies.

### 2.6 Advancements in AI

Artificial Intelligence (AI) is a rapidly advancing field, with new models being developed and tested daily to improve their effectiveness and accuracy. Innovations like DeepSeek-R1, OpenAI's GPT-4 and Google's Bard exemplify this progress, demonstrating remarkable advancements in understanding and generating human-like language. Among the most notable developments in AI are Multimodal Large Language Models (MLLMs) and Large Language Models (LLMs), which represent the cutting edge of artificial intelligence. These models combine deep learning and natural language processing (NLP) to transform healthcare. By utilizing sophisticated neural network architectures, LLMs and MLLMs can comprehend, generate, and analyse human language while processing diverse data types, such as medical images, audio recordings, and textual information. This capability allows for a comprehensive understanding of complex medical scenarios, enabling applications like disease diagnosis, medical report generation, treatment planning, and mental health support. Such advancements highlight the potential of AI to revolutionize healthcare delivery and improve patient outcomes (13). For instance, LLMs such as ChatDoctor and MLLMs such as Med-PaLM M specifically designed to tackle clinical challenges. These models are capable of processing both textual and visual medical data, significantly enhancing decision support systems in healthcare (201, 202). These advanced models excel in a variety of healthcare applications, such as virtual health assistance, automating administrative tasks, and supporting clinical decision-making. They enable a wide range of functionalities, including remote patient counselling, medical question-answering, dialogue summarization, electronic health record (EHR) generation, and clinical health reasoning. By streamlining healthcare operations and facilitating timely interventions, they play a crucial role in enhancing patient outcomes and creating more efficient healthcare systems (13). Despite their transformative potential, deploying Large Language Models (LLMs) and Multimodal Large Language Models (MLLMs) in clinical settings presents several challenges. Developing these models typically requires extensive annotated medical datasets, which are not only costly to acquire but also demand expert-level annotation and raise significant data privacy concerns. Furthermore, the high computational resources needed for training and deploying these models can limit accessibility, particularly for smaller healthcare institutions. Additionally, these models are prone to hallucinations—generating fabricated or inaccurate outputs—and often lack real-time updates, which can compromise their reliability and safety in fast-paced, dynamic clinical environments (203). Addressing these challenges is essential to unlocking the full potential of LLMs and MLLMs in transforming healthcare. Ongoing research is focused on mitigating many of these obstacles. For instance, efforts are being made to create high-quality synthetic datasets, often generated using AI-assisted methods. These synthetic datasets reduce reliance on expensive, expert-annotated medical data, making the development of these models more scalable and cost-effective (201). Additionally, fine-tuning techniques such as reinforcement learning from human feedback (RLHF) and AI feedback (RLAIF) are being employed to align model outputs with clinical expectations and reduce hallucinations (204). Innovations in model optimization, such as parameter-efficient tuning and smaller, specialized models are reducing computational costs, benefiting smaller institutions (205). The future of LLMs and MLLMs lies in real-time learning, enhanced data privacy (using federated learning and encryption), and better integration of diverse data types for more comprehensive insights. Further reducing computational overhead through model compression and energy-efficient training will broaden access, making these technologies feasible for smaller healthcare providers. Crucially, collaboration among AI developers, medical professionals, and policymakers is vital to ensure the development of ethical, user-centred, and clinically effective AI solutions that drive personalized and efficient healthcare (13).

## 3 India's roadmap to digital health

**Figure 7:**
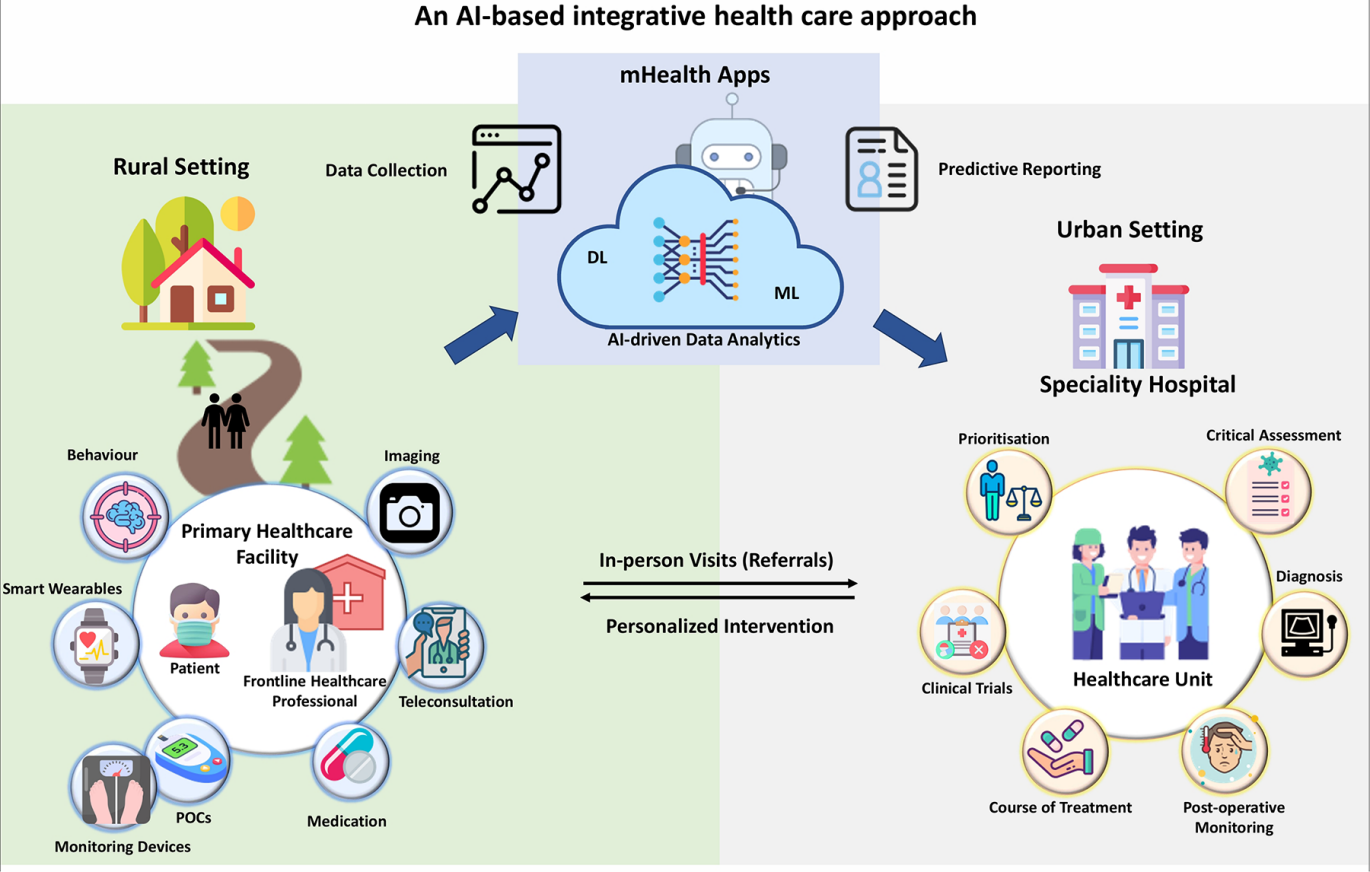
This figure illustrates how technology and training can enable collaboration between urban super speciality centres and rural healthcare professionals. Patient data (clinical reports, imaging, vitals signatures) collected by wearable and other point-of-care (POC) devices can be securely transmitted through cloud-based digital health platforms (mHealth Apps) for AI-driven analysis by urban specialists. A specialized committee can ensure continuous monitoring and timely diagnosis, empowering rural healthcare practitioners with actionable insights for prompt interventions.

Digital health harnesses advanced technologies such as telemedicine, mobile health applications (mHealth apps), electronic health records, and wearable devices to enhance healthcare accessibility, quality, and efficiency. These innovations facilitate remote patient monitoring, virtual consultations, and real-time data sharing, which are essential for overcoming geographical barriers and providing timely medical interventions. In regions with significant rural populations, access to specialized care is often hindered by inadequate infrastructure and a shortage of well-trained physicians. A digital health-based initiative can address this issue by integrating urban super-speciality centres with AI-based technology and training healthcare providers in resource-limited rural centres. This strategy establishes urban hubs for specialized care while empowering rural practitioners with advanced digital health systems. Telemedicine platforms can connect rural patients with urban specialists, ensuring expert care is available without necessitating travel. Mobile health apps support continuous health tracking and personalized health management, enabling patients to take proactive steps in their care. Central to this initiative is the utilization of wearables and point-of-care (POC) devices for remote collection of essential patient data. This data is securely transmitted to cloud-based digital health platforms (mHealth apps) with ultra-high-speed computing capabilities, enabling rapid AI-driven evaluation and analysis by experts at tertiary care urban centres. A dedicated action plan committee, operational around the clock, can monitor this data, conduct critical analyses, and relay prompt diagnoses and actionable insights back to primary healthcare providers in rural centres (Figure 7). This streamlined process facilitates swift and effective personalized interventions, optimizing patient outcomes even in remote areas with limited access to specialized care. For actionable insights on enhancing AI adoption in rural-urban healthcare integration, please refer to Box 3.

Box 3 **Actionable insights for enhanced AI adoption in rural-urban healthcare integration.**

These recommendations aim to bridge the gap between rural and urban healthcare, ensuring equitable access to advanced medical care through AI technologies.

**Table:** 

1. Teaching and Training Programs: ∙ Integrate AI in medical and technical education. ∙ Offer workshops and certification programs for healthcare providers. ∙ Encourage internships and practical training in AI-driven settings. 2. Electronic Health Records (EHR) and Health Portals: ∙ Mandate the use of EHRs for accurate patient data. ∙ Develop secure patient portals for easy access and management of health records. 3. Telemedicine and Remote Monitoring: ∙ Implement telemedicine for remote consultations with urban specialists. ∙ Use wearable devices for real-time health monitoring. ∙ Train healthcare providers and patients on digital health platforms (mHealth Apps).	4. Policy Development and Implementation: ∙ Establish protocols for AI adoption covering data security and ethics. ∙ Provide financial incentives and support for AI integration in rural healthcare. 5. Mobile Health Units and Outreach Programs: ∙ Deploy mobile health units with AI tools for regular check-ups in rural areas. ∙ Educate rural populations about the benefits of AI and telemedicine. 6. Continuous Monitoring and Evaluation: ∙ Establish metrics to evaluate AI interventions. ∙ Create feedback systems for continuous improvement.

## 4 Challenges of adopting AI in healthcare

While AI has the potential to revolutionize cancer research in India, it is not without its challenges and limitations. The adoption of AI in this field is hindered by issues such as regulations (206), reliability of knowledge (207), difficulties in execution (208), its ability to adapt to diverse ecosystems (209), lack of transparency interpretability and data security.

### 4.1 Ethical/regulatory challenges

The rapid evolution of artificial intelligence (AI) technologies often outpaces the development of comprehensive regulatory frameworks, resulting in uncertainties surrounding approval processes, compliance standards, and ethical considerations, particularly in healthcare. The absence of clear regulations often leads to hesitation among healthcare providers and developers in fully embracing these innovations due to concerns about legal liabilities and ethical repercussions.

Globally, organizations such as the World Health Organization (WHO) have launched initiatives like the Global Strategy on Digital Health 2020–2025, which outlines recommendations for establishing AI governance frameworks to promote ethical AI adoption in healthcare (210). In India, however, the regulatory environment for AI in healthcare remains fragmented, hindering the integration of AI-powered tools in critical areas such as cancer diagnosis and treatment. To address these challenges, government initiatives like the National Digital Health Blueprint (NDHB) propose a comprehensive regulatory framework to ensure the ethical use of AI, focusing on data privacy, interoperability, and algorithmic accountability (211). Additionally, the Indian Council of Medical Research (ICMR) has issued ethical guidelines for AI applications in biomedical research and healthcare, emphasizing data security and patient privacy to bridge existing regulatory gaps (212).

Despite these efforts, significant work is still needed to establish robust policies governing AI deployment in healthcare while safeguarding patient rights and ethical practices. International collaborations, such as the AI for Health program by the International Telecommunication Union (ITU) and WHO, aim to support member countries in developing consistent global standards and regulatory frameworks for AI in healthcare (213, 214). These initiatives seek to harmonize international standards, enabling countries like India to adopt global best practices and strengthen their regulatory frameworks.

### 4.2 Data quality

One of the primary challenges in developing effective AI models for cancer care is the lack of standardized, diverse, and inclusive datasets related to cancer health. Training algorithms on inadequate, small, or outdated datasets can introduce biases and reduce the accuracy of outcomes in critical procedures such as cancer detection and therapy. In India, patient records often lack comprehensive details on genetic backgrounds, lifestyle factors, or previous treatments. This scarcity and inconsistency of data hinder the training of AI algorithms, leading to biased and potentially erroneous results. For example, algorithms trained on limited datasets—such as breast cancer biopsy images from a single hospital—may fail to generalize effectively to larger, more diverse populations. Additionally, outdated datasets that do not reflect recent advancements in oncology can compromise the reliability of AI systems. Globally, initiatives like The Cancer Genome Atlas (TCGA), the International Cancer Genome Consortium (ICGC), the Global Alliance for Genomics and Health (GA4GH), and large-scale repositories such as the UK Biobank are addressing these gaps by creating comprehensive and diverse healthcare datasets. These efforts aim to foster interoperability and inclusivity in AI model development, ensuring that algorithms are trained on representative data (37, 215, 216).

India has also recognized the importance of addressing data diversity and inclusivity in healthcare AI. Initiatives like the Indian Cancer Genome Atlas, aim to compile comprehensive cancer-related multi-omics datasets across diverse populations (217). The integration of genomic data into such datasets is being facilitated by organizations like the Council of Scientific and Industrial Research (CSIR) and the Regional Centre for Biotechnology (RCB) through projects such as IndiGenomes and GenomeIndia (218, 219). These efforts, combined with collaborations between global tech companies and healthcare institutions, are critical in reducing biases and ensuring that AI models are inclusive, reliable, and representative of diverse populations. Such initiatives are essential for addressing the healthcare needs of India's heterogeneous population effectively.

### 4.3 Replicability

The replicability of AI findings across healthcare systems is a significant concern. Models trained in one environment often fail to perform consistently in others due to variations in data, infrastructure, and clinical practices. Clinical practice at academic medical centres and community hospitals still differs significantly today, but these differences are decreasing in high-income countries, like the United States, due to the integration of oncology practices by integrated health networks and new businesses that streamline oncology workflows and integrate genomic data, and translate these data into actionable reports (220, 221). In developing nations like India, the pace at which these changes occur depends on several aspects, such as disparities between urban hospitals and rural medical centres. Achieving operational consistency across different geographical locations requires not only protocol standardization but also workforce training in AI tools.

In India, the Ayushman Bharat Digital Mission (ABDM), launched in 2021, aims to create a unified digital health ecosystem (222). By integrating healthcare facilities and standardizing data collection practices, ABDM seeks to reduce discrepancies and improve the replicability of AI systems across the country. Additionally, collaborations between the National Cancer Grid (NCG) and international partners are helping establish uniform oncology practices and enhance the sensitivity and robustness of AI tools in diverse settings (223).

Globally, organizations like the European Commission are driving the development of standardized healthcare protocols through initiatives such as the European Health Data Space, which aims to harmonize healthcare data across EU member states (224). Similarly, the MIT Critical Data Consortium is addressing replicability challenges by developing open-source healthcare datasets and fostering collaborations between academic and healthcare institutions (225). These efforts are crucial for ensuring the consistent performance of AI models across various healthcare settings and geographies.

### 4.4 Infrastructure

The integration of artificial intelligence (AI) into healthcare systems requires substantial investments in infrastructure, workforce training, and public awareness. In India, significant barriers such as limited computational resources and data storage solutions, and the availability of medical equipment and healthcare personnel make the widespread implementation of AI-driven solutions challenging. Initiatives such as the Digital India program and the establishment of Supercomputing Mission Centres aim to address these infrastructural gaps. Furthermore, the Indian Government's recent approval of the IndiaAI Mission, with a budgetary allocation exceeding Rs. 10,000 Crore, represents a significant step towards establishing a robust AI ecosystem in the country (226, 227). Additionally, developing cost-effective alternatives to imaging devices like mammograms, particularly for resource-limited settings, shows great promise in enhancing healthcare accessibility and efficiency. Ministry of Health and Family Welfare, India, has been implementing an action plan for Cancer screening program in rural areas under the Ayushman Bharat scheme. This initiative focuses on strengthening infrastructure, human resource development, health promotion, early diagnosis, management and referral of three common cancers i.e., oral, breast, and cervical (228).

In the private sector, Apollo Hospitals has established India's first AI-driven Precision Oncology Centre in Bengaluru. This centre integrates AI tools to provide personalized cancer care, improve diagnostic accuracy, and optimize treatment planning (121). Such initiatives highlight the growing adoption of AI-enabled healthcare solutions in India and signify a significant advancement in the country's healthcare landscape.

### 4.5 Transparency and interpretability

Additionally, there may be resistance from healthcare professionals due to a lack of familiarity with AI tools or fear of being replaced, leading to challenges in the adoption and utilization of AI in healthcare. A major concern is the lack of transparency and interpretability in AI systems, often referred to as the “black box” problem, where algorithms reach conclusions without providing clear reasoning. This opacity exacerbates trust issues among clinicians and patients, particularly in critical areas like oncology, where clear justifications for medical decisions are essential. Without understanding how an AI system arrives at a specific conclusion, it becomes difficult to trust and act upon its recommendations. This lack of transparency raises ethical concerns and can hinder the clinical adoption of AI technologies. Furthermore, inherent biases in AI models toward specific demographic groups raise additional ethical and practical concerns, especially in a multicultural country like India.

To address these challenges, the Ministry of Electronics and Information Technology (MeitY) has launched initiatives such as the Responsible AI for Social Empowerment (RAISE) summit and the National AI Portal, which aim to promote AI awareness and ethical practices (229). Programs like the AI in Healthcare Certification Program by the All-India Institute of Medical Sciences (AIIMS) and collaborations between the Indian Institute of Technology (IIT) and global AI leaders like Microsoft are pivotal in training healthcare professionals to use AI tools effectively. Research institutions are also focusing on developing explainable AI (XAI) models to ensure that clinicians and patients can understand and trust AI-generated recommendations.

On an international scale, the Global Digital Health Partnership (GDHP), a collaborative effort involving multiple countries, including India, focuses on sharing best practices and resources for AI integration in healthcare (230). These efforts are crucial for building trust, addressing ethical concerns, and ensuring the responsible deployment of AI in healthcare systems.

### 4.6 Misdiagnosis and misuse

The accuracy and consistency of AI systems are heavily dependent on the quality of their training and validation datasets. Due to the inherently predictive and probabilistic nature of AI, these systems can sometimes make incorrect decisions, potentially leading to misdiagnosis, inappropriate treatment plans, or failure to identify critical health conditions, which cannegatively impacting patient outcomes. Incorporating larger and more diverse datasets in training can helpmitigate biases and improve accuracy. Moreover, continuous advancements in AI technology are essential for reducing errors and enhancing predictive performance. While methods exist to detect bias during model training, their effectiveness still requires considerable improvement. Also, continuous validation and rigorous testing of AI systems in real-world scenarios are crucial to identify and rectify errors before deployment, ensuring improved accuracy and reliability.

Deep Learning (DL) methods, such as Convolutional Neural Networks (CNNs), have shown promise inmedical applications, with validation rates ofapproximately 90% for cancer prediction using medical imagery. However, these techniques are often complex and and computationally intensive. For instance, the Convolutional Neural Network (CNN) classifier, used in about 41% of experiments, has demonstrated good performance but demands significant computational resources (25, 231). Despite their potential, the practical application of these models in clinical settings remains inadequately validated. Predictions made by AI models often require verification in a clinical context to support medical experts in making diagnostic decisions. Currently, there are no comprehensive regulations or guidelines to establish legal responsibility when AI systems cause harm or malfunction (232, 233). This underscores the urgent need for well-defined legal frameworks to ensure accountability and enhance patient safety. In the United States, the Food and Drug Administration (FDA) oversees the approval of AI-based medical devices to ensure their safety and efficacy (234–236). Similar regulatory frameworks are needed globally to address the ethical and legal challenges associated with AI in healthcare.

### 4.7 Data security

Addressing the ethical misuse of AI technologies, such as the creation of deepfake medical images or the manipulation of patient data, requires a comprehensive and multi-layered approach to data security and privacy. Strengthening the robustness of AI models through techniques like adversarial training can make them more resilient against manipulation and malicious attacks. Advanced data security measures, such as end-to-end encryption, multi-factor authentication, and secure access controls, are essential to protect sensitive patient information from unauthorized access, tampering, or breaches. Additionally, implementing regular audits, real-time system monitoring, and anomaly detection mechanisms can help identify and mitigate potential ethical abuses swiftly and effectively.

In India, the proposed Digital Personal Data Protection Bill (2023) introduces stringent regulations aimed at ensuring the secure handling of patient data and preventing misuse (237). Additionally, initiatives led by the National Health Authority, such as the Ayushman Bharat Digital Mission (ABDM), focus on establishing robust accountability frameworks for AI-driven healthcare solutions (222, 238). These efforts emphasize transparency, ethical usage, and patient-centric safeguards. Together with global best practices, these measures provide a strong foundation for ensuring that AI technologies are deployed responsibly, fostering trust while minimizing the risks of ethical misuse.

## 5 Conclusion

**Figure 8:**
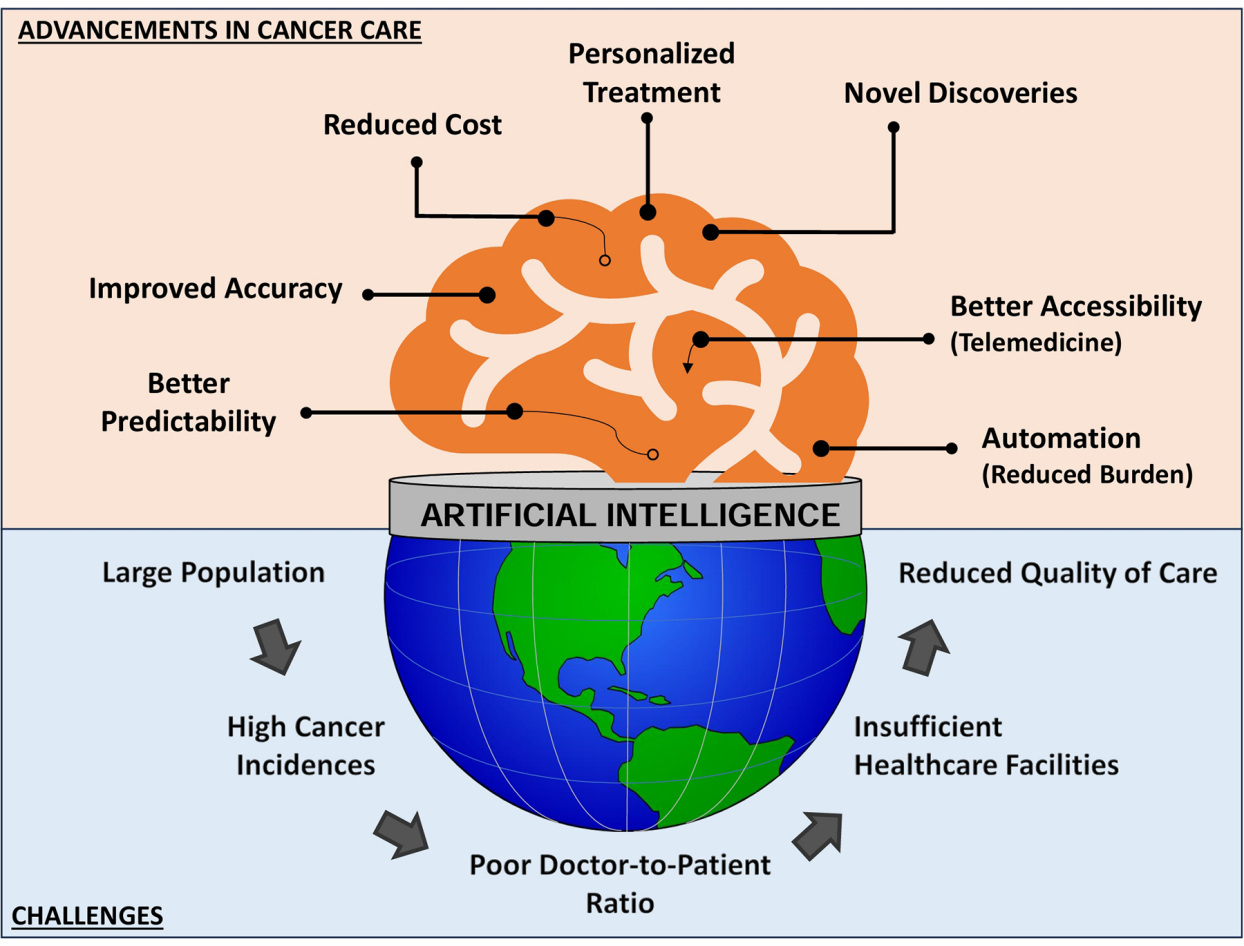
Pictorial representation of challenges encountered by populous countries and how advancements driven by artificial intelligence and digital health can enhance the management of cancer care.

Cancer incidence in India for the year 2022 was estimated at 14,61,427 cases with a rough rate of 100.4/100,000 (239) with about 1 in 9 people expected to develop cancer at some point in their lifetime. This poses an enormous burden for the country. Almost all public healthcare facilities and infrastructure are overwhelmed by this disproportionate number of cases and inadequate resources. Healthcare facilities and healthcare providers (physicians paramedics and nurses) are sparse, particularly in rural and remote areas resulting in healthcare inequities and treatment disparities. Artificial Intelligence can revolutionize cancer care in developing populous nations by filling access gaps, improving healthcare delivery and boosting patient outcomes to better manage the difficulties and engage its large population (Figure 8). AI can leverage an overwhelmed healthcare and still can screen a broader population and intervene early by utilising predictive analytics technology. This review elaborates on the various domains of cancer management including cancer screening, diagnosis, precision treatment, prevention and surveillance using smarter algorithms and predictive tools.

In the future clinical setting, integrating computational input and assistance will become a tangible reality, leading to a significant technological revolution in real-time prediction and diagnosis of human health-related issues. Telemedicine will improve convenience, personalization, and access to specialized care, ultimately leading to better outcomes and a more patient-centred approach to cancer care. Android-based mobile apps and smarter wearables can perform uninterrupted health monitoring and flag any abnormalities at a very early stage. Artificial neural networks and deep learning will serve as optimal decision-making intelligence and evolve continuously, aiding physicians in rapid diagnosis decision-making and exploring treatment regimens.

However, it's essential to understand that AI in clinics only seeks to replace radiologists and other medical professionals partially. Instead, it will function as a novel and potential tool to achieve highly specific treatment performance and identify accurate diagnoses at the highest possible level while maintaining human involvement in final decision-making. As AI technology becomes more prevalent in healthcare, it is crucial to focus on fairness, inclusivity, data security, and adequate training of healthcare personnel. We are at the stage of known knowns (empowering AI in healthcare) but must be cautious of known unknowns (to what extent AI can be a reliable tool in disease diagnosis within its predictive limits) before we step into unknown unknowns (the risk and the ethical turmoil using highly innovative and smarter algorithms may bring in the future). It is best in the interest of humanity to explore the safer and judicious usage of AI for the betterment of society.
